# Mutational analysis of cytoplasmic domain of integrin subunit alpha-1 and its association with periapical wound healing after surgical endodontic treatment

**DOI:** 10.1371/journal.pone.0303627

**Published:** 2024-10-24

**Authors:** Tuba Ghazal, Muhammad Adeel Ahmed, Fazal-ur-Rehman Qazi, Muhammad Mohsin Haider, Sana Naeem, Rizwan Jouhar, Muhammad Farooq Umer, Muhammad Faheemuddin, Veeriah Chowdary Jasthi, Nouman Mughal

**Affiliations:** 1 Department of Community Dentistry, Dr. Ishrat-ul-Ebad Khan Institute of Oral Health Sciences, Dow University of Health Sciences, Karachi, Pakistan; 2 Department of Restorative Dental Sciences, College of Dentistry, King Faisal University, Al-Ahsa, Kingdom of Saudi Arabia; 3 Department of Operative Dentistry and Endodontics, Dr. Ishrat-ul-Ebad Khan Institute of Oral Health Sciences, Dow University of Health Sciences, Karachi, Pakistan; 4 Department of Community Dentistry, Bahria University Dental College, Bahria University Health Sciences Campus, Karachi, Pakistan; 5 Department of Biological and Biomedical Sciences, Aga Khan University Hospital, Karachi, Pakistan; 6 Department of Preventive Dental Sciences, College of Dentistry, King Faisal University, Al-Ahsa, Saudi Arabia; 7 Department of Prosthodontics and Implantology, College of Dentistry, King Faisal University, Al-Ahsa, Saudi Arabia; 8 Department of Oral and Maxillofacial Surgery and Diagnostic Sciences, College of Dentistry, King Fai-sal University, Al Ahsa, Saudi Arabia; University of Puthisastra, CAMBODIA

## Abstract

**Background:**

Numerous studies reported that the healing after surgical endodontic retreatment is influenced by multiple factors which include the genetic profile of the patient, epigenetics, and immune responses. The genes which are primarily responsible for the healing potential in different individuals are those which are involved in the regulation of the cytoskeleton and cellular adhesion which subsequently affects bone deposition and healing. Integrins are cell-surface molecules, possess a key role in the cytoskeleton and cellular adhesion. Integrin Subunit Alpha 1 (ITGA1) is one among the integrin family and helps in regulating the Epidermal Growth Factor receptor (EGFR) pathway, consequently affects proliferation and healing. The objectives of the study were to identify mutations in the cytoplasmic domain of Integrin Subunit Alpha 1 (ITGA1), to assess the expression of activated EGFR, EGFR^Phospho^ and TC-PTP in the periapical wound and to correlate these mutations and expression patterns with periapical wound healing.

**Methods and findings:**

Thirty-seven patients between ages 18–60 years reported chronic apical periodontitis of single-rooted anterior teeth with periapical radiolucency, equal or greater than 4 mm or periapical lesion in an open apex of single-rooted teeth due to trauma were included in the study from 01^st^ June 2018 till 31^st^ October 2019. Patients with persistent radiolucency after primary root canal treatment and endodontic retreatment were kept on follow-up for 3–4 months surgical endodontic treatment was performed in cases with persistent periapical lesions of 4mm or more in diameter. Periapical lesion sample was collected and used for (1) histo-pathological analysis after Hematoxylin & Eosin staining, (2) total DNA extraction for ITGA1 cytoplasmic domain mutational analysis and immunohistochemistry for EGFR and TCPTP. A positive correlation was observed between the expression levels of **EGFR**^**Phospho**^ and the healing of periapical lesions. Moreover, a negative weak correlation was observed between the expression levels of EGFR and TCPTP and the healing of periapical lesions. Out of nine sequences of cytoplasmic domain of ITGA1 which were analyzed, none of them was detected with SNP.

**Conclusion:**

Higher expression levels of EGFR^Phospho^ and lower expression levels of EGFR and TCPTP were associated with patients with good healing potential in periapical area. However, immunohistochemistry scores were statistically insignificant to draw any conclusion.

## Introduction

Periapical granuloma are the most common type of periapical lesions. Periapical lesions are commonly the sequel of irreversible pulpitis and pulpal necrosis [1]. Irreversible pulpitis presents itself as moderate to severe pain that may or may not linger, spontaneous or throbbing pain and/or pain that may be referred to areas other the site of origin. Followed by irreversible pulpitis and pulpal necrosis, apical periodontitis can be either symptomatic or asymptomatic. Symptomatic apical periodontitis presents itself as pain and discomfort on biting while asymptomatic apical periodontitis can be a chance finding on radiograph. Symptomatic apical periodontitis can be acute or chronic as well. Other than periapical granuloma, the lesions usually manifest as periapical cysts and abscess. Histopathologically, periapical granuloma is the granulomatous inflammation of the periapical tissue, mainly comprises of connective tissue, blood vessels and inflammatory infiltrates. Clinically, these lesions are commonly asymptomatic and found on routine investigations. These are presented as a well-defined, homogenous radiolucency in the periapical area of a non-vital tooth.

Periapical lesions possess different etiologies which include immunological, genetic, epigenetic and idiopathic causes. Following pulpal necrosis, once periapical lesion attains chronicity, vascular cellular proliferation of the host tissue is the common reaction in an attempt of repairing the lesion and leads to formation of granulation tissue [2,3]. The pathogenicity of the lesion depends upon a number of factors which include endodontic flora, cellular components of host and molecular mediators including cytokines, growth factors and antibodies [4].

For established periapical lesions, conventional orthograde root canal treatment is the first appropriate choice of treatment. The lesion is then monitored for at least three months and if the tooth is symptomatic and radiolucency is consistent or progressive in nature, then non-surgical orthograde retreatment or surgical retreatment procedures are recommended.

Wound healing is a complex and multifactorial process which has its own dynamics. The process involves a set of several genes that play vital role in healing after injury in human body. According to the study conducted by Garlet et. al, it was hypothesized that the periapical lesion might be an outcome of an imbalance in the expression of these genes [5] ITGA1 is one of these genes belongs to the integrin family of wound healing genes. The key role of ITGA1 is already established in different healing phases of different pathologies in human body. These are also considered as genes, which control the loss of collagen content and ECM adhesion in healing lesions [6,7].

ITGA1 has alpha and beta subunits. Both alpha and beta subunits have external domain, a transmembrane domain and a cytoplasmic tail. This cytoplasmic tail crucially is involved in multiple pathways in healing. Among several target proteins, TCPTP (T-cell protein tyrosine phosphatase) is a ubiquitously expressed protein that ITGA1 interacts with. This interaction activates TCPTP which results in reduced EGFR phosphorylation. Consequently, it inhibits EGFR induced cell proliferation and directly affects wound healing.

Along with genetic and epigenetic factors, there can be numerous factors that play role in healing of periapical lesions and can be considered as confounding variables. These include age, gender, smoking status, and a number of systemic and metabolic conditions like diabetes, vascular diseases, immunocompromised and autoimmune conditions. Moreover, multiple local factors may also delay healing of periapical lesion after surgery which include size of the lesion, size of the surgical window and type of root-end filling.

## Materials and methods

### Patient selection

Thirty-eight participants between age 18–60 years, were selected from patients reporting in the Department of Operative Dentistry, Dow University of Health Sciences from 01^st^ June 2018 till 31^st^ October 2019. The ethical approval was taken by the Institutional Review Board, Dow University of Health Sciences. (IRB-1014/DUHS/Approval/2018). Patients were requested to give written informed consent before initiating the treatment.

The inclusion criteria of the study included patients between 18–60 years of age presenting with chronic apical periodontitis of single rooted anterior tooth/teeth with previously attempted or failed root canal treatment and a periapical radiolucency of 4mm or more and patients presenting with a history of trauma and a periapical radiolucency around the apex of an immature tooth root. The exclusion criteria of the study included the patients presenting periapical lesions of multirooted teeth, periapical radiolucency less than 4mm, or patients with uncontrolled systemic disease, pregnant or lactating patients.

A total of 38 patients meeting the inclusion criteria, were included in the study. Among these, 10 patients were excluded due to lost to follow up or insufficient tissue sample retrieved.

### Endodontic retreatment and tissue retrieval

Initially, a metallic reference marker was attached to the sensor and a preoperative digital radiograph was taken. Non-surgical root canal re-treatment was performed and patients were kept on follow up for 3 months. Patients who presented with persistent or enlarging periapical radiolucency after 3 months, were scheduled and appointed for periapical surgery. Endodontic surgery was performed following standard protocols and periapical tissue was retrieved. The collected tissue was divided into two fragments; one fragment was stored in 10% buffered formalin and sent for histopathological analysis and the other fragment was stored in RNA Later solution and sent for mutational analysis.

### Tissue processing for histopathological analysis

The fragment of the tissue that was stored in 10% buffered formalin was processed for histopathological analysis. The tissue was placed in a tissue processor and treated with increasing concentration of ethanol and then xylol. The tissue is then embedded in paraffin. The paraffin blocks were made and then cut into 5μm sections. These sections were then treated with two sequences of xylol, then decreasing concentration of ethanol, then washed with distilled water and placed in haematoxylin for 3 minutes. The sections were then washed in tap water for 15 minutes and immersed in 70% ethanol, then kept in eosin for 5 minutes. Later, the samples were immersed in absolute ethanol for four times and then in xylol for three times. Lastly, these were covered with cover slips.

Microscopic analysis of these slides was performed using Nikon Eclipse E200 optical microscope (Nikon Instruments Inc., Tokyo, Japan). Microscopically, periapical granuloma was appreciated by the presence of granulation tissue surrounded by fibrous connective tissue and the presence of macrophages, lymphocytes, plasma cells, giant cells, fibroblasts and mast cells. Only those samples which were classified as periapical granuloma, were included in the study.

### Fixation and tissue processing for light microscopy and immunohistochemistry (IHC)

Fixation and processing of samples was done using the standard protocol [8]. Firstly, each sample was fixed in 10% neutral buffered formalin. It was processed in an automated tissue processor (Medite TPC 15). Formalin-fixed paraffin-embedded tissue blocks (FFPE) were prepared in automated paraffin embedding (TES 99 Medite). Sectioning (3–4 μm) was done using a microtome (SLEE 4062). It was smoothed out in warm water bath (46–48°C). It was fixed on coated glass slide labeled with specimen representative number for identification. Deparaffinization was done (in 60°C in an oven for 30 minutes sections followed by three xylene rinses of 5 minutes each). Rehydration was done (in Ethanol immersion for 2 minutes each in 100%, 95%, 80%, and 70% respectively). It was washed under running tap water for 1 minute.

### Hematoxylin and Eosin staining (H&E)

H&E staining was performed as per standard protocols.

### Immunohistochemical staining (IHC) for EGFR, EGFR^Phospho,^ and target protein TCPTP

Immunohistochemical staining for EGFR, phosphorylated EGFR, EGFR^phospho^ and TCPTP was done using following protocol. Each one of mounted slides was incubated in antigen retrieval solution (Cell Marque, USA) in a pre-heated water bath (95–99°C) for 40 minutes. It was let to cool at room temperature for 20 minutes, then washed with distilled water 2–3 times for 2 minutes each. Endogenous peroxidases were inactivated with 3% H2O2 (Hydrogen peroxide) (Cell Marque, USA) for 10 minutes. Two washes in Tris buffer saline with tween (TBST) (Cell Marque, USA) for 2 minutes each. Then it was incubated with diluted primary antibodies (ab52894, ab32430 and ab227916) for 90 minutes at room temperature followed by incubation with peroxidase-labeled polymer complex for 35 minutes. It was then washed three times with TBST and then incubated with secondary antibodies at room temperature for 15 minutes and washed again. Afterwards, substrate chromogen solution was applied (Envision), 3–3’-diaminobenzidine (DAB) substrate was applied for 5 minutes and washed with distilled water. Then, counterstaining was done with Harri’s hematoxylin. It was washed again under tap water for 10 minutes and decolorized with 1% acid alcohol. Slide was dehydrated with increasing concentrations of Ethanol as 70%, 80%, 95%, and 100% and cleared with changes of immersion in xylene/phenol solution (1:1) for 2 minutes each. Lastly, coverslips were mounted using DPX (BDH).

### Tissue analysis and scoring of immunohistochemistry

The immunoreactive scores (IRS) were calculated for EGFR, EGFR^phospho^ and our target protein TCPTP, as a product of positive cells’ percentage with staining intensity. The percentages of positive cells were scored from 0–4 as: 0 = 0%, 1 = <10%, 2 = 10–50%, 3 = 51–80%, 4 = >80%. The staining intensity was measured as 0 = no staining, 1 = weak staining, 2 = intermediate staining, 3 = strong staining. The IRS was grouped into 0 = no expression, 1–4 = low expression, 5–8 = moderate expression, and 9–12 = strong expression [9].

## Results

### Baseline characteristics and clinical features

After periapical surgery was performed, a total of 37 samples were obtained. Out of these, 9 were excluded due to loss of follow-up or insufficient sample tissue. The baseline characteristics of the patients are presented in Table 1. Majority of patients fall in the age group 18–30 years i.e. 85.7% (n = 24), followed by 31–40 years i.e. 10.7% (n = 3) and 3.6% (n = 1) in 41–50 years of age group. Most of the patients were males 64.3% (n = 18) and 35.7% (n = 10) were females.

**Table 1:** Baseline characteristics of the patients (n = 28).

Characteristics	N	%
**Age (years)**		
18–30	24	85.70
31–40	3	10.70
41–50	1	3.60
**Gender**		
Male	18	64.30
Female	10	35.70

At the time of initiation of the surgical endodontic procedure, all clinical features indicating pulpal and periapical pathologies such as the presence of pain, sinus tract formation, tenderness to palpation and percussion, and tooth discoloration were observed at high percentages (Table 2). Among them, 53.6% (n = 15) patients reported pain while 46.4% (n = 13) also reported swelling in the particular tooth/teeth pre-operatively. Moreover, 67.9% (n = 19) patients reported that the tooth/teeth were tender to palpation while 53.6% (n = 15) patients reported that periapical area or the tooth crown/root were tender to percussion as well. In 53.6% (n = 15) patients, there was presence of sinus tract pre-operatively. Tooth discoloration was observed in 57.1% (n = 16) patients. Patients with marked tooth discoloration i.e. 57% (n = 16) were advised to go for internal bleaching or porcelain fused metal crown to improve esthetics (Table 2).

**Table 2:** Pre-operative clinical signs and symptoms.

Signs	N	%
**Pain**		
No	13	46.40
Yes	15	53.60
**Swelling**		
No	15	53.60
Yes	13	46.40
**Sinus tract**		
No	13	46.40
Yes	15	53.60
**Tenderness to palpation**		
No	9	32.10
Yes	19	67.90
**Tenderness to percussion**		
No	13	46.40
Yes	15	53.60
**Tooth Discoloration**		
No	12	42.90
Yes	16	57.10

The post-treatment follow-up was carried out after 12 months. Among these, successfully recorded were 24 cases that showed complete or partial bone healing on the periapical radiograph alongside a complete resolution of clinical symptoms such as pain, swelling, and sinus tract formation. On the contrary, 05 cases revealed uncertain healing on radiography and were still tender to percussion and palpation. Only one case was recorded as complete clinical and radiographic failure showing no signs of bone healing or resolution of any of the pre-operative clinical features.

### Mutational profile of cytoplasmic domain of ITGA1

**Fig 1:**
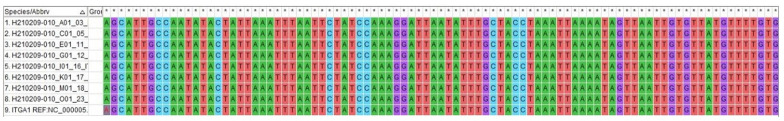
Mutational analysis of the cytoplasmic domain of ITGA1 (spanning nucleotides 1165–1179 KIGFFKRPLKKKMEK).

Nine sequences of cytoplasmic domain of ITGA1 were analyzed. Out of these nine sequences, none of them found to have any SNP (Single-nucleotide polymorphism) Fig 1. Obtained sequences were visualized & Trimmed by using BIOEDIT and aligned against the reference sequence (NC_000005.10) downloaded from NCBI by using MEGA7 with clustalW method.

### Identification of protein expression and its pattern for EGFR, EGFR^Phospho^, and TCPTP using immunohistochemistry

Expression analysis of EGFR, EGFR^Phospho^ and target protein TCPTP was performed using immunohistochemistry as discussed above in Chapter 3. Out of 28 cases, EGFR expression was low in 78.6% of cases (n = 22), while in 17.9% cases (n = 5) there was a moderate expression observed, and only 3.6% i.e. n = 1, presented with high expression of EGFR. However, EGFR^Phospho^ presented altered expressions as in only 21% of cases (n = 6) there was a low expression observed, while 39.3% cases (n = 11) presented with moderate expression and exactly 39.3% (n = 11) presented with high expression. Surprisingly, the expression of our target protein TCPTP seems quite similar to EGFR as in 75% of cases (n = 21) the expression was low, while 17.9% (n = 5) presented with moderate expression, and in only 7.1% cases (n = 2) the expression of TCPTP was high (Table 3).

**Table 3:** Expressions of EGFR, EGFR^Phospho^, and TC-PTP (n = 28).

Expressions	N	%
**EGFR**		
Low	22	78.60
Moderate	5	17.90
High	1	3.60
**EGFR** ^**Phospho**^		
Low	6	21.40
Moderate	11	39.30
High	11	39.30
**TC-PTP**		
Low	21	75.00
Moderate	5	17.90
High	2	7.10

The expression patterns of EGFR, EGFR^Phospho^ and target protein TCPTP were also observed and recorded as either focal or diffuse on immunohistochemistry. The EGFR expression was focal in 42.9% of cases (n = 12) and diffuse in 57.1% of cases (n = 16). However, the expression of EGFR^Phospho^ was focal in 7.1% of cases (n = 2) and diffuse in 92.9% of cases (n = 26). Contrarily, the expression of target protein TCPTP appeared focal in 78.6% of cases (n = 22) and diffuse in 21.4% of cases (n = 6) (Table 4).

**Table 4:** Expression pattern of EGFR, EGFR^Phospho^ and TC-PTP (n = 28).

Expressions	N	%
**EGFR**		
Focal	12	42.90
Diffuse	16	57.10
**EGFR** ^**Phospho**^		
Focal	2	7.10
Diffuse	26	92.90
**TC-PTP**		
Focal	22	78.60
Diffuse	6	21.40

### Clinical evaluation of healing

Periapical Index (PAI) scores were assessed pre-operatively; immediate post-operatively, 6 months and 12 months follow-up. At the time of pre-operative assessment and examination 85.71% (n = 24) cases were found to hold PAI score 4 while 14.28% (n = 4) cases were at PAI score 3. As mentioned in the inclusion criteria, only those cases included in the study which possessed persistent or progressive lesion, so all the cases 100% (n = 28) showed PAI scores 4 on the radiographs taken immediate post-operatively. At 6 months follow up, a decent number of cases showed clinical and radiographic healing. Among these, 7.14% (n = 2) cases were recorded as PAI score 1, while 46.42% (n = 13) cases were at PAI score 2. However, 32.14% (n = 9) were assessed as PAI score 3 while 14.28% (n = 4) cases were at PAI score 4 (Table 5).

**Table 5:** Comparison of PAI scores; pre-operative, immediate post-operative, post-operative follow up of 6 months and 12 months (n = 28).

PAI Scores	Pre-Operative		Immediate Post-Operative		6 months Follow up		12 months Follow up	
**1**	0	0	0	0	2	7.14	10	35.70
**2**	0	0	0	0	13	46.42	12	42.90
**3**	4	14.28	0	0	9	32.14	5	17.90
**4**	24	85.71	28	100	4	14.28	1	3.60

After 12 months of follow up, periapical radiographs were taken and healing was assessed again. Among these, 35.7% cases (n = 10) that presented with complete healing (i.e. no clinical signs and symptoms and complete resolution of radiolucency), fall in PAI score 1. However, 42.9% of cases (n = 12) that reported with incomplete healing (i.e. no clinical signs and symptoms and periapical radiolucency smaller than that of preoperative radiograph) were recorded as PAI score 2. However, 17.9% of cases (n = 5) presented with uncertain healing (i.e. absence of clinical signs and symptoms and periapical radiolucency smaller in size with some cystic changes observed) were recorded as PAI score 3 Lastly, 3.6% of cases i.e. only one case presented with clinical signs and symptoms postoperatively hence, considered as unsatisfactory healing and PAI score 4. (Table 6).

**Table 6:** Clinical evaluation of healing (n = 28).

Scores	N	%
PAI scores		
1	10	35.70
2	12	42.90
3	5	17.90
4	1	3.60

### Histopathological evaluation of healing and its correlation with EGFR, EGFR^Phospho^ and TC-PTP

**Fig 2:**
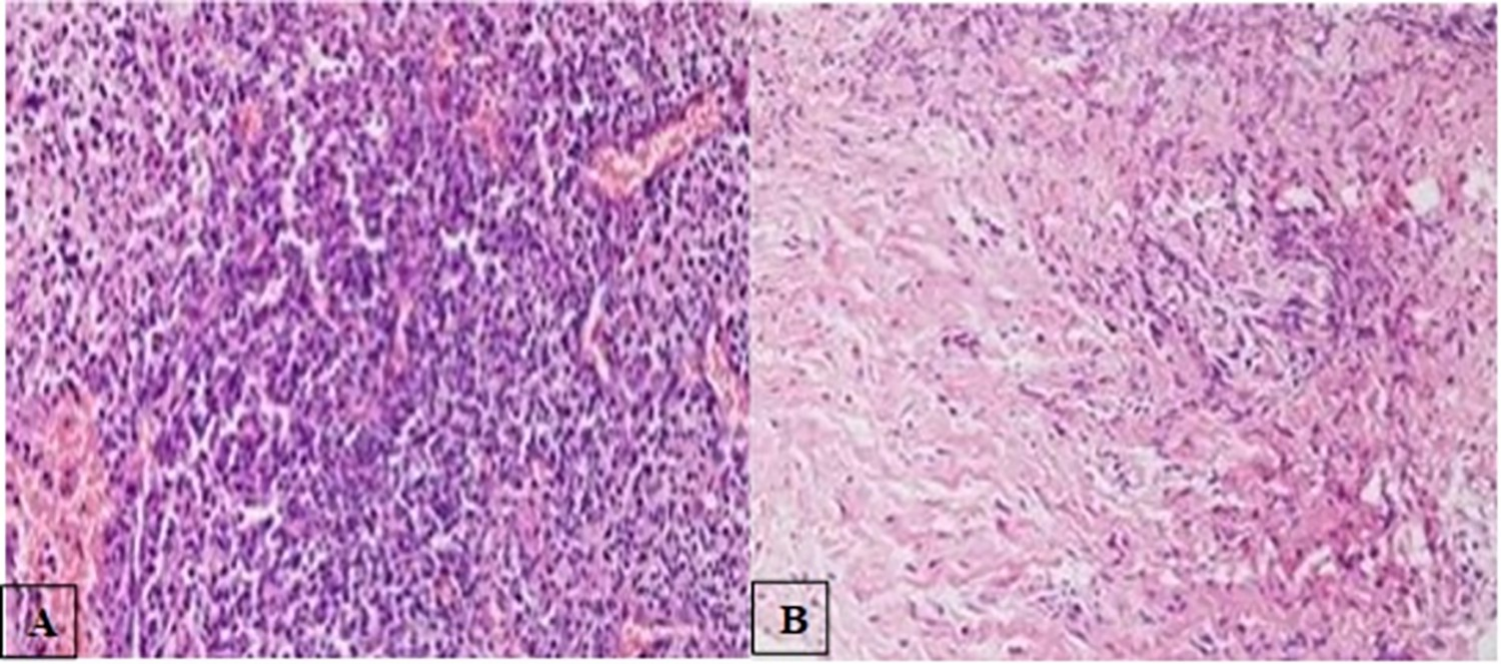
A. The histopathological image of healing group (H & E) original magnification x400). B. The histopathological image of non-healing group (H & E) original magnification x400).

The tissues from periapical granuloma were analyzed on histopathological grounds. The histopathological image from the healing group as seen in Fig 2A shows granulation tissue with prominent blood vessels along with significant infiltration of inflammatory cells comprised of lymphocytes, plasma cells and histiocytes. However, the histopathological image from the non-healing group (Fig 2B) demonstrates fibrous connective tissue with significant number of fibroblasts and fewer inflammatory cells and blood vessels.

For the ease of understanding and interpretation, PAI scores 1 and 2 are considered as ‘Healing’ cases (n = 22) while PAI scores 3 and 4 are considered as ‘Non-healing’ cases (n = 6).

Periapical wound healing was correlated with baseline characteristics of patients i.e. age and gender, and with expression analysis of EGFR, EGFR^Phospho^ and TCPTP, using Chi-square test. The first age group i.e. 18–30 years patients showed noteworthy healing potential. 21 patients from age group 18–30 years presented as healing cases, while only 3 patients presented as non-healing cases.

In our limited data, there were only 3 patients from the age group 31–40 years, among them, 1 patient presented as healing case while 2 patients presented as non-healing cases. The only patient who belongs to the age group 41–50 years presented with a PAI score of 3, hence falls under the non-healing category.

Among the patients who showed low expression of EGFR, 86.4% (n = 19) were with good healing potential while 13.6% (n = 3) were among non-healing cases. Out of 5 cases that showed moderate expression of 60% (n = 3) were healing cases while 40% (n = 2) were non-healing cases. The only case which showed high expression of EGFR was under the non-healing category. (Table 7) The patients who showed the low expression of our second marker EGFR^Phospho^ 83.3% (n = 5) were among healing cases while 16.7% (n = 1) were non-healing cases. Out of 11 cases of moderate expression of EGFR^Phospho^ 90.9% (n = 10) were healing cases while only 1 patient i.e. 9.0% showed non-healing potential. 63.6% (n = 7) of patients with good healing potential showed higher expressions of EGFR^Phospho^ while 36.4% (n = 4) were non-healing cases. (Table 7).

**Table 7:** Correlation of EGFR, EGFR^Phospho^ and TC-PTP with wound healing (n = 28).

Variables	PAI scores		p-value
	Healing (n = 22)	Non-healing (n = 6)	
	n (%)		
**Age (years)**			
18–30	21 (87.5)	3 (12.5)	0.015^†^
31–40	1 (33.3)	2 (66.7)	
41–50	0	1 (100.0)	
**Gender**			
Male	14 (77.8)	4 (22.2)	0.999^ǂ^
Female	8 (80.0)	2 (20.0)	
**EGFR**			
Low	19 (86.4)	3 (13.6)	0.064^†^
Moderate	3 (60.0)	2 (40.0)	
High	0	1 (100.0)	
**EGFR** ^**Phospho**^			
Low	5 (83.3)	1 (16.7)	0.282^†^
Moderate	10 (90.9)	1 (9.0)	
High	7 (63.6)	4 (36.4)	
**TC-PTP**			
Low	17 (81.0)	4 (19.0)	0.440^†^
Moderate	3 (60.0)	2 (40.0)	
High	2 (100.0)	0	

†p-value was calculated using Chi-square test

ǂ p-value was calculated using Fisher exact test.

The third marker i.e. the target protein, TCPTP showed an incredibly dissimilar pattern. Among the patients which showed low expression of TCPTP, 81.0% (n = 17) were healing cases while 19.0% (n = 4) were non-healing cases. Out of 5 patients who exhibits a moderate expression of TCPTP, 60.0% (n = 3) were of good healing potential while 40.0% (n = 2) were non-healing cases. The only 2 cases which exhibit high expression of TCPTP were among the healing category and there was no case of high expression among non-healing cases. To find out the correlation between EGFR, EGFR^Phospho^ TCPTP, Spearman’s rho correlation test was applied. The values of correlation coefficients for EGFR and EGFR^Phospho^ were 0.231 and 0.247, respectively which indicates a weak correlation of these markers with wound healing. However, TCPTP showed strikingly dissimilar results. The value of correlation coefficient for TCPTP was -0.009 which indicates weak negative correlation between the expressions of TCPTP and wound healing. (Table 8).

**Table 8:** Correlation of EGFR, EGFR^Phospho^ and TC-PTP with wound healing (n = 28).

PAI scores		
	R	p-value*
**EGFR**	0.231	0.237
**EGFR** ^**Phospho**^	0.247	0.206
**TC-PTP**	-0.009	0.963

*p-values have been calculated using Spearman’s rho correlation test.

## Discussion

Surgical and non-surgical treatment options are intended to ensure complete periapical healing. However, in cases where the lesions fail to heal and persist despite following all the treatment protocols, the reasons should be considered on genetic grounds. With the background of what is already known about the cytoplasmic domain of ITGA1, EGFR, and EGFR^Phospho^ expressions in healthy oral tissues, and its effects on the expression of TCPTP, this study was conducted to evaluate the potential role of the cytoplasmic domain of ITGA1 in periapical wound healing and to assess the role of any mutations found in this particular domain of Integrin Subunit Alpha 1.

In the present study with regards to gender and age, a higher number of participants were males (n = 18) and belonging to the younger age group of age 18–30 years (n = 24) (Table 1). The males included in the study were 64.3% (n = 18) while females were only 35.7% (n = 10).

According to the results of the study after 12 months follow up participants presenting in the younger age group of 18–30 years showed the most potential for healing as they recorded the highest number of PAI healing scores 1 and 2 (complete and partial healing). Only one participant in the age group of 31–40 years showed a PAI score of 1 whereas no participant in the age group 41–50 years showed a PAI score of 1 or 2. Although in the present study insignificant differences (p = 0.073) were observed between the three age groups due to the limited sample size of the study and unequal participants distribution among the different age groups. Nonetheless, this observation may signify that the potential for periapical healing decreases with age. This pattern of more healing potential is consistent with other studies as well [10,11]. The main reason attributed to this better healing potential of younger patients directly relates to the aging phenomenon. As with age, there are osteoporotic changes in the alveolar bone which results in decreased bone density and increased bone fragility. Moreover, the number of stem cells and bone vascularity is also diminished. Hence, the alveolar bone becomes thinner and more porous, this slows down bone repair [12].

Although age plays a crucial role in healing, underlying genetic factors are also involved. In the present study, we did not manage to recover good-quality DNA from granuloma samples, since most of the tissue was comprised of necrotic tissue. Therefore, the tissue collection process and storage protocols need to be improved to get good quality DNA. Continuing this experiment on a larger scale, more samples are being collected and stored for further analysis.

In the past, healing was generally assessed in multiple studies but there was no study on the healing potential of patients in the periapical granuloma. Hence, the results are quite interesting in terms of EGFR and EGFR^Phospho^ related to healing.

Before assessing the localization of EGFR and EGFR^Phospho^ and TCPTP in cases of periapical granuloma, it is crucial to establish its role in healthy control tissues. According to the literature, the collagen-binding α1β1 acts as a negative regulator of epidermal growth factor receptor (EGFR) signaling through the activation of protein TCPTP and activates it after binding to collagen. The cytoplasmic tail of α1is selective in its interactions hence interacts with TCPTP and activates it after cell adhesion to collagen. This activation results in reduced EGFR phosphorylation [13].

Although the values of EGFR and EGFR^Phospho^ are not statistically significant there were 77.2% (n = 17) cases with moderate to high levels of EGFR^Phospho^ in patients with good healing potential (Table 5A). However, 86.4% (n = 19) cases with low levels of EGFR showed good periapical healing. Even though the p-values are not statistically significant, the trend can be observed and appreciated that lower expression levels of EGFR and higher expression levels of EGFR^Phospho^ are associated with good periapical healing. In the future, this should be further investigated on a larger sample size to get conclusive results. According to the present study, 75% of cases (n = 21) showed lower levels of nuclear TCPTP in patients with good healing potential which may indicate that lower nuclear levels of TCPTP promote healing.

The assessment of radiographic healing was performed via taking a digital periapical radiograph using paralleling technique. The pre and post-treatment PAI scores were recorded for all patients. Before the apical surgery was performed, the PAI score for all participants was 4 as previously mentioned in the inclusion criteria of the study. Post-treatment 12 months follow-up showed that 22 (78.6%) out of 28 cases included in the study showed a PAI score of 1 or 2 indicating complete or partial bone healing signifying a high success rate of treatment. Five cases showed uncertain healing (PAI score of 3) and only one case was recorded as failure or unsatisfactory healing showing no periapical bone healing (PAI index of 4) (Table 4B). Similar observations to the present study were reported by Von Arx T et al, they performed a 10-year follow-up study on 119 MTA-filled apecoectomy teeth [12]. They reported a high success rate of 81.5% of healed cases. Another study by Kruse C. *et*. *al*. performed a 6-year follow-up to evaluate periapical healing in apecoectomy cases with and without MTA as a retrograde filling material [14]. They reported higher percentages of healed cases (86%) in the MTA treated teeth as compared to its counterpart (55%).

The present study has failed to detect any strong association between expression levels and healing scores. This could be due to the relatively small sample size and lack of colocalization study which would provide better insight into the association of these antibodies with healing.

Although the relatively small sample is insufficient to provide a statistically significant association between the levels of EGFR and TCPTP, however low cytoplasmic expression of EGFR and moderate to high cytoplasmic expression of EGFR^Phospho^ in the majority of cases of progressive periapical healing might indicate that cytoplasmic expression of EGFR^Phospho^ is favorable to healing.

The present study also evaluated the correlation of periapical healing with certain patient characteristics such as age and gender, and expression of epigenetic proteins such as EGRF, EGRF (P) proteins, and TC-PCP (Table 3). Similarly, insignificant healing potential (p = 0.820) was observed to gender differences with males scoring higher percentages PAI scores 1 and 2 in contrast to females. Similar observations to the present study have been reported by Von Arx T et al, they found no significant association between periapical healing and parameters such as age, gender, type of MTA used [12]. On the contrary, another study by the same authors [15], concluded that factors such as lesion size, retrograde filling material, and female gender significantly influence the healing outcome of periapical surgeries. A study by Caliskan MK *et*. *al* reported similar findings to the present study [16]. They concluded that parameters such as gender, age, tooth type and location, and histopathological status of the periapical lesion had no significant influence on the outcome of apical surgery.

## Conclusion

Although immunohistochemistry scores were insignificant to draw any conclusion for EGFR, EGFR^Phospho,^ and TCPTP expressions associated with periapical healing, a particular trend was observed with the lower expression levels of EGFR and TCPTP and higher expression levels of EGFR^Phospho^ in patients with good healing potential in periapical area.

## Supporting information

S1 Raw data (XLSX)
